# Translational Clinical Research: Use of Allogeneic Platelet-Rich Fibrin (PRF) for Wound Regeneration in Two Small-Sized Dogs

**DOI:** 10.3390/ani15030367

**Published:** 2025-01-27

**Authors:** Carla S. Soares, Isabel R. Dias, Luís C. Barros, Pedro P. Carvalho, Maria dos Anjos Pires

**Affiliations:** 1 Animal and Veterinary Research Centre (CECAV), AL4AnimaLS, Department of Veterinary Sciences, School of Agricultural and Veterinary Sciences, University of Trás-os-Montes e Alto Douro (UTAD), 5000-801 Vila Real, Portugal; carlasoares.medvet@gmail.com (C.S.S.); idias@utad.pt (I.R.D.); 2 VetLamaçães Veterinary Clinic, 4715-303 Braga, Portugal; luiscmbarros23@gmail.com; 3 Vetherapy—Research and Development in Biotechnology, 3030-509 Coimbra, Portugal; pedro@vetherapy.co

**Keywords:** allogeneic, small-sized dogs, hemoderivative, immunogenicity, PRF therapy, regenerative, wound healing

## Abstract

Platelet-rich fibrin (PRF) is a recent regenerative biomaterial applied in clinical wound healing. This study aims to evaluate the safety and efficacy of allogeneic PRF therapy as a regenerative methodology for treating skin wounds in two small dogs. A Miniature Pinscher and a mixed breed dog weighing 3.6 and 9.5 kg, aged 5 and 10 years, with extensive skin wounds implicating significant tissue loss and with abundant yellow exudate were allocated to this study. Each allogenic PRF treatment was derived from screened donors and consisted of grafting PRFs at the wound recipient area. Nevertheless, the blood group of the donor or recipient was not considered. The wound from case 1 received three PRF treatments during the initial two weeks of treatment, and case 2 received four PRF treatments, followed by single weekly ones. The utilization of PRFs was suspended as soon as relevant wound contraction and epithelization were observed. Both animals received systemic antibiotics prescribed at the emergency room by the first clinician. The wounds were cleaned using sterile saline only. Two weeks later, the lesions had reduced by more than 80%. All lesions exhibited aesthetic and uneventful healing. No rejection, necrosis, or infection signs were observed. PRF acted as a regenerative biomaterial, forming vascularized granulation tissue, followed by epithelization and wound resolution.

## Simple Summary

The present work aims to report the safety and efficacy of allogeneic platelet-rich fibrin (PRF) therapy as a regenerative methodology for treating skin wounds in two small-sized dogs. A 5-year-old Miniature Pinscher male dog, weighing 3.6 Kg, and a 10-year-old mixed breed female dog weighing 9.5 kg, presenting with extensive skin wounds with significant tissue loss were allocated to this study. Each allogenic PRF treatment was derived from screened canine donors, but the blood groups of both donor and recipient were not considered. PRF treatments consisted of grafting allogenic PRF at the wound recipient area. The wounds were cleaned using sterile saline only during PRF grafting treatments, and no antiseptics were used in any of the treatments. Two weeks later, the lesions had reduced by more than 80%, and from the second week onwards, all wounds were treated approximately once a week, instead of the conventional bandage change every 3 to 4 days. Furthermore, no clinical signs of rejection, necrosis, or infection were observed. PRF acted as a regenerative biomaterial, forming vascularized granulation tissue, followed by epithelization and wound closure. All lesions exhibited aesthetic and uneventful healing in a 4- and 6-month follow-up period, for cases 1 and 2, respectively.

## 1. Introduction

This paper reports the treatment of two naturally occurring wounds in two small-sized dogs, a male Miniature Pinscher and a mixed breed female dog weighing 3.6 and 9.5 kg, respectively, using allogeneic platelet-rich fibrin (PRF). PRF therapy involves the use of a blood derivative and is defined as an effective and cost-effective biological treatment for wound healing. PRF therapy has been increasingly reported in human medicine. There are scarce reports regarding allogenic PRF therapy in the veterinary area, but treatments are being trialed. Autologous PRF therapy has been reported in both human and veterinary settings but may have some limitations in certain patients, especially those where blood sampling is not possible [1,2,3]. PRF is defined as a biocompatible and biodegradable hemoderivative consisting of a natural fibrin scaffold containing elevated platelet and leukocyte aggregates associated with the release of high concentrations of bioactive growth factors (GFs) and cytokines [4,5]. These GFs have a central role in the regulation and modulation of cellular activities, through the activation of intracellular signaling pathways, leading to the proliferation, differentiation, or apoptosis of local cells, and even to the secretion of other proteins by intervenient cells, with paracrine effects [6]. The proliferation of fibroblasts, endothelial cells, and keratinocytes occurs during the skin repair, embedded in a complex molecular crosstalk established between several resident and recruited cellular elements within the injured region [7]. In addition, other studies have recognized the antimicrobial, anti-inflammatory, and analgesic effects of PRF therapy [8,9].

This report aims to describe the therapeutic performance of allogeneic PRF therapy as a grafting technique for the treatment of traumatic-occurring skin lesions in small dogs involving substantial tissue loss, where the autologous methodology was not viable. The two cases reported corroborate allogenic PRF therapy in the medical treatment of two wounds in a real clinical scenario in two small dogs.

## 2. Materials and Methods

This study was approved by the Ethics Committee of the University of Trás-os-Montes E Alto Douro. The donor’s and recipient’s owners provided consent for blood collection and PRF administration.

The recipients of PRF therapy were two dogs, weighing 3.6 and 9.5 kg: a Miniature Pinscher and a mixed breed dog, aged 5 and 10 years, respectively, with extensive skin wounds implicating substantial tissue loss. Moreover, the lesions presented intense yellow exudate. The treated dogs were allocated for allogenic PRF therapy because the owners declined the first therapeutic proposal. In case 1, surgical wound cleaning and debridement were proposed (additional to the other treatment performed in another clinic), allowing the lesion to heal with regular bandage changes (approximately every 2–3 days). The owner did not accept this recommendation. In case 2, the first therapeutic recommendation was to perform a skin graft, in a second surgical procedure, with wound management using bandage changes until a healthy bed of granulation tissue could allow skin grafting. Nevertheless, the owner declined due to the potential for graft complications.

The donors of PRF biomaterial had an indoor lifestyle, were screened via an immunofluorescence antibody test, vaccinated for *Leishmania* spp., received regular internal and continuous external deworming, and were up to date with vaccinations. The blood types of the PRF donors and recipients were not considered, and therefore donors were not selected based on the blood group of the recipient. They did not receive any vaccine or medication during or in the 15 days before their selection as donors. The canine donors were clinically evaluated before the donation and were in good health.

Each PRF clot was produced using an aseptic technique, as described in former works [5]. Briefly, 5 mL of blood required to produce one PRF clot was drawn from each canine donor’s jugular vein after hair clipping and antiseptic cleaning of the puncture area. The blood was transferred into a sterile polypropylene tube, 57 × 15.3 mm, without clot activator, and was immediately centrifuged at 3000 rpm (584× *g*) for 10 min at room temperature. The blood was left to rest inside the tube for 60 min after the centrifugation step. Using sterile materials, the PRF clots were removed from the tubes, and the red fraction was removed, obtaining the fraction of interest of the PRF (the white portion) ready to be grafted topically into the wound.

All of the skin lesions were irrigated with a sterile 0.9% saline solution and dried with a sterile gauze immediately before each PRF application. Each PRF treatment consisted of the application of newly produced PRFs at the recipient area, ensuring contact between the platelet and leukocyte-rich area of the clot and the lesion. At the end of the PRF application, a closed bandage was applied using a sterile petroleum jelly gauze and dry bandage, maintaining the clot at the recipient wound area. The number of PRFs applied at the recipient site depended on the size of each wound. One PRF clot was applied for each 2.5 cm^2^ wound surface, approximately.

The PRF treatment was performed twice and three times in the first week for cases 1 and 2, respectively, changing to once a week afterward. The utilization of PRF was suspended as soon as granulation tissue covered the wound bedding, associated with relevant wound contraction and the initiation of epithelization. The bandaging procedure was consistently performed using a sterile petroleum jelly gauze and dry bandages until complete wound closure was achieved. During the period between the suspension of PRF therapy and complete wound closure, only dry gauze was applied to cover the wounds. The application of dry gauze was suspended when a solid and small crust was visible, indicating an advanced stage of recovery. Although bandages are generally not required during the mature phase, they were applied in these cases to prevent visualization by the owners and to reduce the effects of possible scratching by the animal, which could delay healing, especially in friable wounds that are prone to bleeding.

The same observer was responsible for the evaluation of the photographs of the wound during the healing process. The wound area image was obtained by ImageJ^®^ software (version Image J^®^: 2.1.0/1.53c, Bethesda, MD, USA) using the photographs of each patient at each time point. The percentage of wound contraction (%WC) was calculated using the formula [(Wound Area at day 1 − Wound Area at Specific Time point)/Wound Area at day 1] × 100. If the wound exhibited two distinct areas at a certain time point, both areas were added to provide information with respect to the wound’s total area. The software was used to calculate the percentage of wound contraction only. The software did not evaluate the presence of granulation tissue. The results were expressed as the median and interquartile range (IQR). Wound depth was not considered. Both patients were re-evaluated 4 and 6 months after wound closure (cases 1 and 2, respectively).

### 2.1. Case 1

The male dog presented a large laceration on the left anterior thoracic region, with the exposure of muscular layering. The patient was initially assisted in another clinical center and, according to the owner’s description, was sedated, the extended laceration surgically dressed, and a drain tube was placed. Two days after the trauma, the animal owner sought another clinic and the animal was observed by the present team: the wound suffered a suture dehiscence and drains were removed.

Three days after the trauma, without the use of anesthesia or sedation, the wound measuring 16.12 cm^2^ was profusely irrigated with sterile saline solution and the first PRF treatment was performed. The patient was medicated with clindamycin 11 mg/kg PO q 24 h for 15 days, and carprofen 2 mg/kg PO q 24 h for 5 consecutive days (prescribed in the first attending clinic and, therefore, maintained). A wound culture was not performed as the dog had already received systemic antimicrobial therapy. The dog received methadone 0.3 mg/kg IV q 12 h during the first 24 h of hospitalization, being replaced by tramadol 3 mg/kg PO q 12 h for 5 days (during the first 48 h of hospitalization, the animal was administered SC).

### 2.2. Case 2

The female dog was assisted after suffering a road traffic accident, presenting a large laceration on the hindlimb with exposure of the lateral collateral ligaments of the tarsus and tissue loss at the tarsus level. The patient presented pain and had a unilateral pubis fracture, which was medically stabilized. The wound was treated with irrigation using sterile saline solution and protected with a closed bandage in the emergency consultation.

Two days later, with the patient monitored and stabilized, a surgical intervention for the correction of the skin laceration was performed. The complete surgical closure of the lesion was impossible due to the significant tissue loss and exposure of the ligaments.

The PRF therapy was initiated on the first postoperative day, four days following the initial trauma. The lesion was edematous and with fibrin deposition, presenting an area of 6.22 cm^2^. The patient was medicated with amoxicillin–clavulanic acid 20 mg/kg PO q 12 h for 10 days, and meloxicam 0.1 mg/kg PO q 24 h for 5 consecutive days (administered IV during hospitalization, implemented by the emergency team and, therefore, maintained). The patient received methadone 0.3 mg/kg IV q 12 h during the first 48 h of hospitalization and tramadol 3 mg/kg PO q12 h for 5 days (administered after hospitalization).

## 3. Results

### 3.1. Individualized Wound Assessment

#### 3.1.1. Case 1

**Figure 1:**
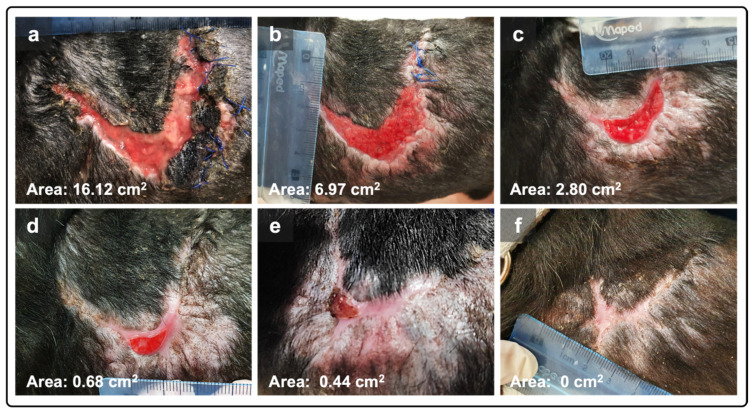
The lesion from case 1, located in the left anterior thoracic area. (**a**) The initial wound before the implementation of the PRF therapy (day 1); (**b**) wound healing after the first PRF application (day 6); (**c**) wound reduction at the third and last PRF therapy application (day 10); (**d**) on day 17, one week after the last PRFs’ application; (**e**) wound aspect at day 23; (**f**) complete wound closure achieved 31 days after initial PRF application.

The PRF canine donor was an 8-year neutered male Labrador Retriever, weighing 38 kg, and PRFs were grafted. The initial surface area of the infected wound was 16.12 cm^2^, at day 1 (D1). After one week of PRF therapy, the wound had a dimension of 6.67 cm^2^, reaching an area of 2.80 cm^2^ and 0.68 cm^2^ on days 10 and 17, respectively (Table 1). Three PRF-grafting treatments and two treatments without PRF (dry gauze) were performed until complete wound closure, achieved one month after the first PRF treatment (Figure 1). The wound treatments were secured using an elastic tubular net bandage around the body.

#### 3.1.2. Case 2

**Figure 2:**
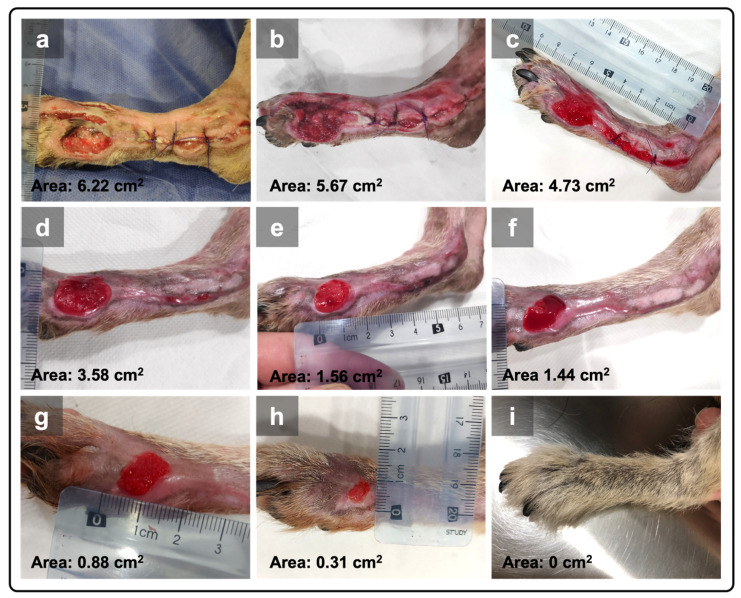
The lesion from case 2, located on the right pelvic limb. (**a**) The wound before starting PRF therapy (day 1); (**b**) initial wound after the first grafting of the PRFs into the lesion (day 4); (**c**) after two applications of PRFs (day 7); (**d**) wound after three applications of PRFs (day 12); (**e**) at the time of the last PRF application (day 19); (**f**) after five PRF treatments (day 26); (**g**) at day 33; (**h**) at day 38, after PRF application was suspended; (**i**) complete closure was documented on day 47, and the skin and fur had a normal appearance 2 months later.

The PRF-canine donor was a 1-year-old female Golden Retriever dog, weighing 27 kg. This lesion received three PRF-grafting treatments in the first week, followed by once-a-week treatment until complete resolution (Table 1). On day 12 after the treatment initiation, the wound surface was reduced to 3.58 cm^2^, recording an area of 1.44 cm^2^ at day 26 and 0.88 cm^2^ after 1 month of PRF therapy. After 47 days of treatment with a total of five PRF applications and three without PRF (dry gauze), complete wound closure was achieved (Figure 2).

### 3.2. Clinical Evaluation of the Performance of PRF Therapy

The allogeneic PRF treatments were well tolerated, with no relevant signs of inflammation, except those inherent to the physiologic wound repair process. Relevant signs of inflammation were elevation of wound edges and turgid peripheric area associated with typical clinical signs (such as swelling, redness, pain, and loss of function). Ischemia or necrosis were not documented in the peripheral area or at the wound bedding.

Regarding allogeneic PRF as a hemoderivative material obtained from screened canine donors but without blood typification tests, their ability to induce granulation tissue formation was confirmed, as observed within the lesions after each subsequent PRF application. Moreover, we did not record any adverse reactions such as severe inflammation or necrosis. The allogenic PRF therapy had the same results as the autologous methodology [10].

**Figure 3:**
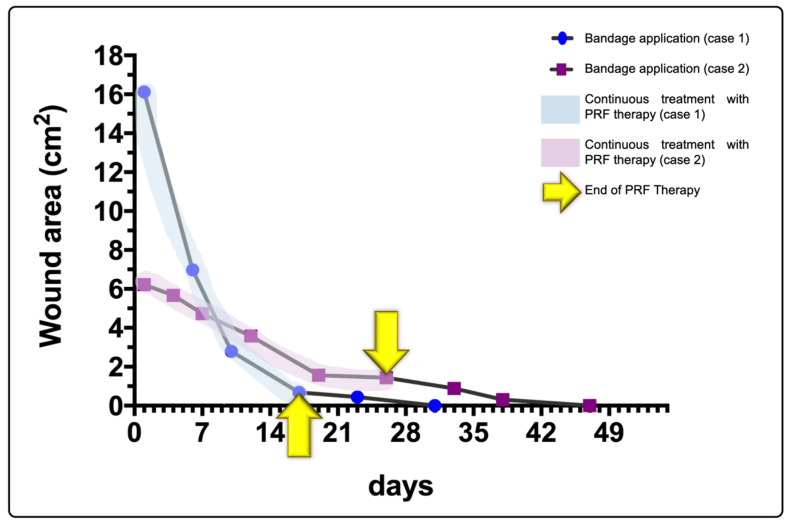
PRF therapy protocol and healing progress of the canine wounds (cases 1 and 2), addressed with allogeneic PRF therapy. A substantial slope is observed in both cases in the early stages of the treatments (until day 20), indicating a significant reduction in the lesion area.

Statistical analysis showed an 80% reduction in the wound area within the first 20 days of PRF treatment, with a significant decrease in the wound area observed in the early stages of treatment (Figure 3) when PRF therapy was actively applied (regarding both the number of PRFs grafted and the number of PRF treatments). Early islet epithelialization was also observed in both wound beds during this period (detected on days 10 and 7 for cases 1 and 2, respectively). This was most likely facilitated by the PRF biomaterial. PRF therapy also reduced the presence of both fibrin and exudate without the need for mechanical debridement. Furthermore, completely healed wounds were achieved. This period corresponds to major PRF treatments actively applied, as stated in Table 1.

Skin crust formation and wound contraction were progressively apparent in both cases. The patients tolerated the repeated wound manipulation involving the PRF-grafting and the bandage changes well without the use of topical analgesics. The lesions recorded aesthetic and uneventful healing without wound recurrence or local alteration for at least 4 months after allogeneic PRF therapy.

## 4. Discussion

PRF has been recently described as a medical treatment for wound management due to its regenerative potential in wound healing [9,11,12,13,14]. PRF is outlined as a biocompatible and biodegradable biomaterial used in platelet therapy, composed of a natural fibrin scaffold containing elevated amounts of platelets and leukocytes, which can release high concentrations of growth factors (GFs) and cytokines [4,5]. PRF acts as a temporal protein-releasing biomaterial, able to release bioactive proteins with important functions in tissue regeneration [15]. These GFs and cytokines are fundamental to activating the molecular pathways, promoting the repair of skin injury and overcoming tissue loss, stimulating the healing by second intention repair [7]. This could explain the possible regenerative effect of the PRF clots, supporting a potential benefit in second-intention healing. PRF is a highly reproducible technique, with no involvement of expensive equipment [5].

In the veterinary context, interest in platelet-based regenerative interventions begins to arise. Autologous PRFs for wound healing have been recently applied in humans and canines, both with infrequent descriptions [10,16,17]. Less commonly, successful allogeneic PRF treatments have also been described in dogs and donkeys [1,18]. Xenogeneic application has been described using canine-sourced PRF clots without adverse effects [3,19]. These were grafted directly into naturally occurring wounds of domestic cats with co-morbidities such as immunodeficiency virus, anemia, and pancreatitis [3].

This report aimed to describe the therapeutic performance of allogeneic PRF therapy, obtained from screened canine donors, as a grafting technique for the treatment of traumatic-occurring skin lesions in small dogs. The autologous methodology was not feasible in the two dogs presented here for several reasons, such as the debilitation caused by trauma suffered by recovering animals, low body weight, and/or the administration of anti-inflammatory medication, which is reported to have a negative impact on the efficacy of platelet therapy, which could affect the clinical outcomes of PRF therapy [20].

The allogeneic administration of platelet-derived products constitutes an advantage over autologous approaches in cases where medical conditions do not allow patient blood harvesting or when blood collection is not advised (e.g., low-volume donors/small-sized donors, uncooperative or unhealthy animals) [21]. Nevertheless, allogeneic therapies carry the risk of transmissible diseases, requiring a rigorous screening of the donors, always considering the geographic prevalence of blood-borne diseases [22]. Separately, donors should be predominantly housed with strict adherence to the vaccination schedule recommended by the World Small Animal Veterinary Association, and with the anti-parasitic recommendations of the European Scientific Counsel Companion Animal Parasites.

The immunogenicity of allogeneic platelet-based formulations such as PRF and platelet-rich plasma (PRP, another well-known platelet-based hemoderivative) was not demonstrated in human clinical descriptions regarding allogeneic therapy. No systemic or local adverse immunological effects were reported using allogenic platelet-based hemoderivatives, applied without considering their blood typification or cross-match checks [23,24,25].

Cross-contamination/bacterial contamination is minimal in PRF manufacturers due to its minimal manipulation processing, whereas for clinical use, it must always be obtained through strict aseptic techniques [26]. The PRFs used in the present study were obtained from screened collaborative canine donors, living indoors, in close contact with the owners, and therefore under strict prophylactic control and aseptic PRF grafting technique executed by the same clinician using an aseptic technique.

The statistical analysis showed a wound area reduction of 80% within the first two weeks of PRF treatment and when PRF therapy was actively applied (regarding both the number of PRFs grafted and the number of PRF treatments), suggesting a direct regenerative effect of the PRF clots. Furthermore, regarding these two cases, from the second week onwards, all wounds were treated approximately once a week, although it is recommended that dressings be changed every 3 to 4 days [19]. Additionally, no antiseptics were used during an of the treatments. Furthermore, no clinical signs of infection were observed in the two wounds addressed: the exudate and fibrin deposition decreased throughout the treatments, as did local edema.

Additionally, the latest in vitro studies suggest the potential antimicrobial action of platelet therapy [9,11,12,13,14]. Both dogs received systemic antibiotics: case 1 for 15 days and case 2 for 10 days. The wounds were managed for more time, for 31 and 47 days, respectively, without antibiotic coverage, and no infection signs were recorded. The dogs received systemic antibiotics prescribed at the emergency room, as advocated by current clinical guidelines/recommendations [20,21,22]. Reflecting on these features, the reports of these two case studies do not allow any assumption regarding the possible antibacterial effect of PRFs.

Recent human trials reported a decrease in patients’ pain, possibly associated with the anti-inflammatory mechanisms of the PRFs [27]. Observing the two cases documented here, both dogs tolerated the repeated wound manipulation involving PRF grafting, but it is important to note a possible analgesic effect due to the pharmaceutical drugs administrated. Clinical studies using control groups and an objective pain scoring system would be required to assess the analgesic effects of PRF on wounds.

The most important goal of PRF therapy stated in this case report was the reduction in the visits to the clinic and the reduction in the frequency of bandage changes. Bandage changes and simultaneous wound treatment on standard medical treatments are required every 3–4 days until wound closure is achieved, always considering the lesion type as an aspect [19]. Both cases had undergone surgical wound treatment before PRF therapy, but both failed: case 1 was clinically infected and suffered dehiscence, and in case 2, complete wound closure was not possible due to severe tissue loss. Effectively, the wounds would naturally heal by the second intention. Still, the application of PRF therapy allowed this physiological step with less wound manipulation and visits to the clinic considering the assessment time points to be properly photographed. In addition, the clinical conditions of the two animals were not the same, as different antibiotics were used, making it impossible to conclude whether the PRF, the antibiotics, or the animal’s immune system itself, or all together, were responsible for the complete wound recovery.

## 5. Conclusions

PRF therapy is a biologic regenerative strategy suitable for medical wound management in veterinary practice. PRF clots are applied directly into the wound as a grafting technique and act as a regenerative promoter. It also provides an alternative technique when a second healing intention is required for various reasons (economic restraints, increased anesthesia risk when surgical debridement is required, among others). PRF is a blood-derived biomaterial that effectively promotes physiologic wound closure, reducing the need for wound manipulation and veterinary visits. Allogeneic PRF therapy is safe and effective in the treatment of traumatic skin lesions in small-sized dogs where the autologous methodology is not viable, demonstrating no immunization risk. Allogeneic PRF should be obtained from screened canine donors.

Furthermore, antiseptics were not used during the treatments, indicating that PRF therapy can benefit the environment in veterinary wound care. Further studies with control groups are needed to fully evaluate the potential benefits of PRF in canine wound management.

## Figures and Tables

**Table 1:** Wound characterization and treatment protocol at the baseline (day 1).

Case	Time Point Day ^+^	Wound Area (cm^2^)	Number of PRFs Applied	Clinical Evaluation	Duration of Treatment and Follow-Up Period
1	1	16.12	4	Visually infected wound, with the presence of abundant yellow exudate (purulent) and fibrin deposition. Detachment of edges and subcutaneous tissue, with marked tissue edema.	31 days Last follow-up: 4 months later
	6	6.97	3	Presence of moderate serohemorrhagic exudate, with less tumefaction. Granulation tissue occupying the lesion bed and absence of wound edge detachment. No fibrin deposition.	
	10	2.80	2	Significant wound contraction and presence of exophytic highly vascularized granulation tissue. Less exudate and epithelization detected.	
	17	0.68	0	Skin contraction and epithelization ongoing. Vestigial exudate present in the bandage.	
	23	0.44	0 *	Crust formation and ongoing wound contraction. No exudate present.	
	31	0.00	0 *	Complete wound closure.	
2	1	6.22	3	Yellow exudate (purulent) in the edges and moderate fibrin deposition. Lesion with significant tissue loss, with ligament and muscular layers exposed, and fibrin deposition. Marked edema and detachment of edges and subcutaneous tissue.	47 days Last follow-up: 6 months later
	4	5.67	3	Intense serohemorrhagic exudate and a focal yellow exudate within the wound (purulent focus present). Reduction of wound edges detachment, but edema still present. Detection of granulation tissue.	
	7	4.73	3	Presence of intense highly vascularized granulation tissue specially in the initial deeper area. Reduction of exudate production, associated with significant wound contraction. Wound edges detachment not present, but mild edema observed. Absence of fibrin deposition. Epithelization islets present in the lesion’s borders.	
	12	3.58	2	Progressive wound contraction, with the increasing of highly vascularized granulation tissue. Reduction of exudate production, but mild edema still witnessed.	
	19	1.56	1	Wound contraction, with closure of proximal area. Epithelization on going. Vestigial exudate.	
	26	1.44	0 *	Presence of exophytic highly vascularized granulation tissue in the initial deeper area. Epithelization ongoing and progressive edge contraction. Subtle crust formation.	
	33	0.88	0 *	Epithelization and contraction ongoing. Less reddish wound bed initial deeper area. Crust formation.	
	38	0.31	0 *	Crust formation and wound contraction.	
	47	0.00	0 *	Complete wound closure.	

Legend: * No PRFs were applied. Only a closed bandage was applied using a sterile petroleum jelly gauze and dry bandage; ^+^ The timepoints correspond to PRF treatments or closed bandage; the only moments were when the lesions were evaluated, photographed, and manipulated.

## Data Availability

Data is contained within the article.
